# Topography and Predictitve Value of Enlarged Perivascular Spaces in Patients with Cognitive Impairment beyond Aneurysmal Subarachnoid Hemorrhage

**DOI:** 10.1002/alz.094734

**Published:** 2025-01-09

**Authors:** Li Gong, Xueyuan Liu

**Affiliations:** ^1^ Shanghai Jiaotong university school of medicine affiliated Tongren hospital, Shanghai, Shanghai China; ^2^ Tongren hospital, Shanghai Jiaotong university school of medicine, Shanghai, Shanghai China

## Abstract

**Background:**

To explore the correlation between the topography of EPVS and cognitive impairment after aSAH.

**Method:**

Patients clinically diagnosed as aSAH by DSA and CT; Head magnetic resonance imaging was performed between 1 week and 1 month after onset, combined with clinical and neuroimaging variables to assess the incidence of hydrocephalus and delayed cerebral ischemia after aneurystic subarachnoid hemorrhage. Follow‐up was performed at 3 months, and the patients' prognosis and cognitive function were evaluated by mRS and the Montreal Cognitive Assesement (MoCA), respectively. The clinical characteristics of aSAH patients with EPVS <10 and EPVSV10 in basal ganglia and centrum semioval were compared, and a binary Logistic regression model was used to study the severity of EPVS and its correlation with DCI, subacute hydrocephalus, poor prognosis and cognitive impairment.

**Result:**

BG‐EPVS predominance pattern (BG‐EPVS > CSO‐EPVS) was more common in the aSAH group (53.8%) than in other primary SAH patients without aneurysm(15.8%). A total of 159 patients completed 3‐month MoCA assessment, of which 63 (39.6%) were diagnosed with cognitive impairment (MoCA<22). EPVS >10 (no matter CSO or BG) was associated with unfavorable functional outcomes at 3 months. BG‐EPVS >10 linked to subacute hydrocephalus and DCI, but not with cognitive impairment after adjusting for established predictors. In contrast, CSO‐EPVS>10 predicted worse cognitive function after adjustment for established variables.

**Conclusion:**

CSO‐EPVS is associated with cognitive impair beyond aSAH, but not with subacute hydrocephalus and DCI, suggesting distinct lymphatic drainage and mechanism after an attack of aSAH.

